# Prevalence of Osteonecrosis of the Femoral Head in High-Risk Male Patients with Severe COVID-19 Treated with High-Dose Corticosteroids: A Prospective Cohort Study Using Screening MRI-How Many Have Been Left Behind?

**DOI:** 10.3390/diagnostics16101466

**Published:** 2026-05-12

**Authors:** Nicola Guindani, Mario Gaffuri, Pietro Andrea Bonaffini, Clarissa Valle, Alessandro Caruso, Greta Carioli, Francesca Fenili, Rosalia Zangari, Sandro Sironi, Federico Chiodini, Claudio Carlo Castelli

**Affiliations:** 1 Orthopaedics and Traumatology Unit, Regional Health Care and Social Agency Papa Giovanni XXIII, Piazza OMS 1, 24127 Bergamo, Italy; fchiodini@asst-pg23.it (F.C.); cccastelli@libero.it (C.C.C.); 2 School of Medicine and Surgery, University of Milano–Bicocca, Piazza dell’Ateneo Nuovo 1, 20126 Milano, Italy; pbonaffini@asst-pg23.it (P.A.B.); cvalle@asst-pg23.it (C.V.); acaruso@asst-pg23.it (A.C.); ssironi@asst-pg23.it (S.S.); 3 Orthopaedics and Traumatology, Moncucco Clinic, via Soldino 5, 6900 Lugano, Switzerland; 4 Department of Radiology, Regional Health Care and Social Agency Papa Giovanni XXIII, Piazza OMS 1, 24127 Bergamo, Italy; 5 FROM Research Foundation–ETS, Papa Giovanni XXIII Hospital, Piazza OMS 1, 24127 Bergamo, Italy; gcarioli@fondazionefrom.it (G.C.); ffenili@fondazionefrom.it (F.F.); rzangari@fondazionefrom.it (R.Z.)

**Keywords:** osteonecrosis of the femoral head, corticosteroids, COVID-19, long-COVID, screening, magnetic resonance imaging

## Abstract

**Objectives**: The association between osteonecrosis (ON) and Coronavirus Disease of 2019 (COVID-19) was reported early during the pandemic. ON of the femoral head (ONFH) is particularly problematic, as it may destroy the joint and lead to arthroplasty, although early diagnosis and treatment might mitigate its progression. The aim of the present study was to quantify the prevalence of symptomatic and asymptomatic ONFH in patients with severe COVID-19 treated with high doses of corticosteroids during the first pandemic wave. **Methods**: For this prospective, observational, monocentric cohort study, patients were selected according to the risk factors described for severe acute respiratory syndrome coronavirus in 2002–2004 (SARS-1): young males (<61 years old), high cumulative cortisone doses (≥2 g), severe disease, and followed up clinically and with magnetic resonance imaging. ONFH was classified with the ARCO classification. **Results**: Out of 1944 patients admitted for COVID-19 from 23 February to 21 May 2020, 856 of 1944 were treated in ICU; 30/1944 were selected according to the inclusion criteria and 27 of 30 were enrolled. The mean age at admission was 54 years (range, 42–60). The mean dose of cumulative cortisone was 6.25 g (range, 2–16). A total of 4/27 (15%) patients had ONFH; only 2 of 4 (50%) were symptomatic, including 1 with multiple ON of major joints. **Conclusions**: A high-risk cohort of patients with COVID-19 and high doses of corticosteroids had a 15% rate of ONFH, and 2 years after the event, 50% of them were asymptomatic. For those patients, relying solely on clinical evaluation would risk underestimating ONFH, potentially influencing treatment and outcomes. Moreover, other joints might develop ON. The data collected in the present study can be considered for the management of long-COVID. The association between severe COVID-19, high doses of corticosteroids and ONFH suggests the need for focused clinical and magnetic resonance imaging, considering the high rate of asymptomatic patients.

## 1. Introduction

The association between osteonecrosis (ON) and coronavirus disease of 2019 (COVID-19), caused by Severe Acute Respiratory Syndrome Coronavirus 2 (SARS-CoV-2), was reported early during the pandemic [1,2,3,4,5]. So far, ON in COVID-19 exhibits most characteristics of other numerous diseases whose pathophysiology and treatment involve inflammation and high doses of corticosteroids [6,7,8].

The well-known causes of ON are corticosteroids therapy, alcohol abuse, smoking, and a broad group of pathologies definable as blood dyscrasias, which have the common denominator of a higher risk of intravascular occlusion (e.g., sickle cell disease, polycythemia vera, metabolic disorders like Gaucher’s disease) [9,10]. Among all the possible locations of ON, the osteonecrosis of the femoral head (ONFH) is particularly problematic. Due to the high mechanical strain of this joint, ONFH may rapidly destroy the joint and lead to arthroplasty. In addition, regardless of etiology, ONFH is the most frequent location of ON [8], and about 50% of patients with ONFH have been treated with corticosteroids [8,11]. In early stages, ONFH is usually asymptomatic [12], and the most sensitive method for its identification and classification is magnetic resonance imaging (MRI) [8,12,13,14,15,16]. However, it is estimated that only about 20% of ONFH cases are diagnosed in the early asymptomatic stage [12].

Early diagnosis and treatment might mitigate the progress of ONFH but are crucial to identify ONFH when the femoral head is still salvageable; unfortunately, at this stage ONFH is usually still less or even asymptomatic. Hence, there is only a narrow opportunity for hip preservation surgery before femoral head collapse, and screening for early detection might reduce the incidence of hip arthroplasty in young patients [8,12,13,14].

The only data available at the time of the COVID-19 pandemic rested on the experience of the SARS-1 epidemic (caused by Severe Acute Respiratory Syndrome Coronavirus nr 1: SARS-CoV-1), dating back to 2002–2004 [17]. According to data from the SARS-1 outbreak, patients treated for a severe respiratory disease developed ONFH in up to 40% of cases. Although the association is well described, it is not clear whether the virus itself is an independent causal factor or if ON is only a side effect of the steroid therapy [11,18,19]. Recently, some authors reported an additive effect between COVID-19 and corticosteroids on ONFH but even with more than 5 years data supporting the relationship between COVID-19 and ONFH, this issue remains incompletely resolved [15,20,21,22]. The aim of the present study was to quantify the prevalence on hip magnetic resonance imaging (MRI) of both symptomatic and asymptomatic ONFH in patients with severe COVID-19 treated with high doses of corticosteroids during the first pandemic wave and in one of the global hotspots regions [23].

## 2. Materials and Methods

This is a prospective observational monocentric cohort study. The primary outcome is to measure the prevalence of ONFH in patients treated for severe COVID-19 and with high doses of corticosteroids, including both asymptomatic and symptomatic cases.

The inclusion criteria were chosen to select patients with an expected higher probability of ONFH from the population treated at the COVID-19 referral center during the first wave of the pandemic, considering the etiologic classification criteria of ARCO on ONFH [15]. At that time there were no reports on this topic; therefore, we referred to data from SARS-1 [19,24]. The inclusion criteria were as follows: admission between 23 February and 21 May 2020; confirmed PCR-positive diagnosis for SARS-CoV-2; age between 17 and 66 years at diagnosis; male sex; treated for severe COVID-19 with a cumulative equivalent doses of cortisone > 2 g (or >0.32 mg methylprednisolone); length of hospital admission exceeding 25 days; and agreeing to participate in the present study by signing informed consent. At the beginning of the pandemia there was no PCR test for SARS-CoV-2 available for clinical practice [25]. As the reliability of clinical and radio-logical diagnosis of COVID-19 is questionable [23,26], patients who had only a clinical diagnosis were excluded from this study. For a more practical computation and reporting, data in the present study are presented as equivalent of Methylprednisolone (1 mg Methylprednisolone = 6.25 mg Cortisone) [27].

**Figure 1:**
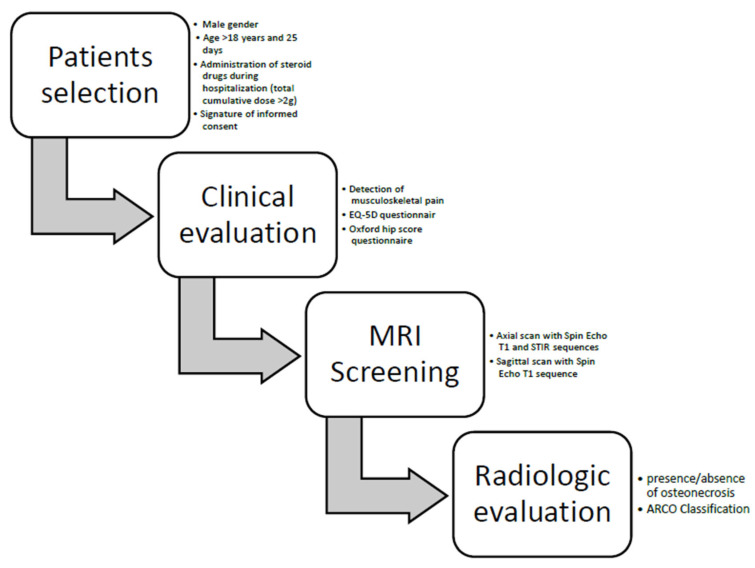
Flow diagram illustrating patients’ selection and screening. Legend: MRI = magnetic resonance imaging, Spin Echo = Spin Echo sequence, T1 = longitudinal relaxation times sequence, STIR = Short-TI Inversion Recovery sequence. ARCO = Association Research Circulation Osseous.

Exclusion criteria were incompatibility and/or contraindication MRI, co-pathologies involving cortisone-intake or at risk for ONFH, previous surgery or existing pathologies affecting both hips, and refusal to participate. The flow diagram illustrating patient selection and screening is reported in Figure 1.

This study was conducted according to the Declaration of Helsinki criteria and followed good clinical practice. This study and subsequent amendments (Protocol Nr 134/2021) were approved by the local ethics committee on 15 June 2021. Every patient gave formal consent prior to participating in this study.

In accordance with the motions of both statisticians and the local ethical committee, this study was parametrized according to the data from SARS-CoV-1 [28]: at that time, no data was available for ONFH and SARS-CoV-2. As the main outcome was the prevalence of ONFH, a control group was omitted in the study design. According to the ethical committee, the outcome (prevalence) could not be modified by any control group, and inference was not planned in any case. Given the expected prevalence of ONFH in the general population and the sample size, a control group would have lacked the cost-effectiveness and ethical basis for the present study (Protocol Nr 134/2021).

### 2.1. Data Parameters

The hospital database and medical charts were searched; relevant data were retrieved and recorded pseudo-anonymously using a dedicated electronic case report form (eCRF). The included patients underwent a clinical evaluation and an MRI at follow-up. ONFH was classified according to the ARCO system [13,14]. Clinical status was quantified using the EQ-5D-5L [29] and the Oxford Hip Score [30]. If a patient reported musculoskeletal symptoms in an area other than the hip, an MRI scan of the involved bone(s) and joint was performed in addition to the planned hip MRI.

### 2.2. MRI Scan

MRIs of the hip were acquired on either a 1.5 Tesla or a 3 Tesla magnet with a dedicated surface coil and with the patient in a supine position. The standard hip protocol included axial and pure coronal longitudinal relaxation time weighted (T1) and short tau inversion recovery (STIR) images covering pelvic bones and proximal femurs. No contrast medium was injected.

### 2.3. Statistical Analysis

A frequentist approach was chosen [31]. Assuming nominal α- and β-errors of 5% and 20%, respectively, an expected prevalence of ONFH in the general at-risk population of 10%, an expected rate of 40% in the sample [28] and a drop-out of 10%, a sample size of 26 patients was obtained.

For the primary outcome, which was descriptive, the use of parametric versus non-parametric statistical tools was determined after performing the Anderson–Darling tests; consequently, the results were described respectively with percentage (%) or 95% confidence intervals (95% CI). The present study was not designed to perform statistical inference; consequently, it has neither been formally performed nor attempted: the variables concerning ONFH and no-ONFH outcomes were plotted and their trends observed. Statistical analysis and plots were performed with Excel—Microsoft 365^®^, Microsoft Corporation, US.

## 3. Results

### Patient Enrollment

According to the hospital charts and databases, during the first pandemic wave, 1944 COVID-19 patients were admitted to our institution and 30 of 1944 met the inclusion criteria [23]. Of these, 2 of 30 refused to participate for personal reasons, and 1 of 30 one was excluded because he had previously been treated with corticosteroids for thrombocytopenic purpura, leaving 27 patients (53 hips) included in the present study. The demographic and clinical characteristics of the enrolled patients with and without ON following COVID-19 infection, also including comorbidities and cardiovascular risk factors, severity of COVID-19, duration of ICU stay, and ongoing chronic therapies at the time of admission, are summarized in Table 1. All enrolled patients completed the study (drop-out 0%).

**Figure 2:**
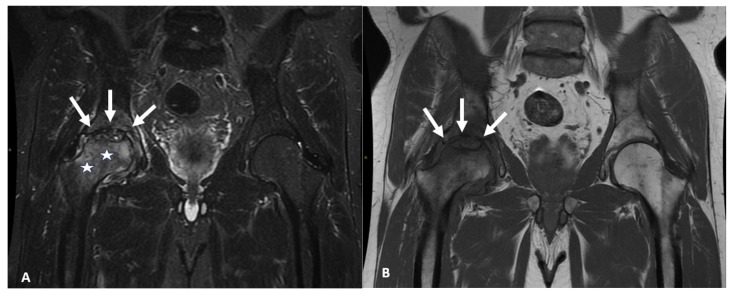
(**A**): STIR coronal view of a 50-year-old male, after severe COVID-19, 42 days in ICU and 1315 mg of methylprednisolone in 45 days. At the time of MRI, the patient was symptomatic (right groin pain after a few meters walk). Typical appearance of femoral head ON on the right side (arrows), associated with locoregional bone marrow edema of the neck-trochanteric region (stars). (**B**). T1 coronal view of the same patient of Figure 1, which shows partial collapse of the right femoral head (arrows).

During the first wave of the pandemic, 1944 patients were admitted for COVID-19 and 856 of 1944 were treated in ICU at our Institution. Of the 27 patients included in the present study, 5 of 27 patients (19%; 95 CI% 0.063–0.381) had MRI signs of ON. A total of 4 of 27 (15%; 95 CI% 0.063–0.381) had ONFH and 1 of 27 (4%) had talus ON without ONFH. Two patients out of 4 (50%) with ONFH were symptomatic; they had bilateral involvement, and one of these two also had multiple ON at the level of major joints (bilateral hip, knee, shoulder, and ankle joints). These two patients with ONFH and symptoms complained of groin, thigh, and buttock pain (Figure 2). The MRI findings of patients with ON are summarized in Table 2.

The median age at the time of hospitalization was 54 ± 4 years (IQR: 42–60), 54 years (IQR 48–55) for those with ON and 54 years (IQR: 51–57) without ON. The median age at follow-up was 56 ± 4 years (IQR: 44 to 63). Patients with ON spent 47 days in the ICU (IQR: 40 to 48 days) and patients without ON 19 days (IQR: 12 to 40), whilst the mean admission time was 67 days (IQR: 64–128) and 63 (IQR: 28–91), respectively. Among them, 15 of 27 patients (56%) were receiving therapy for co-pathologies at the time of admission, and all patients who tested positive for ON with the MRI belonged to this subgroup.

The treatment protocols and indications arising from trials results changed rapidly at that time, so different patients received different corticosteroids during their in-hospital stay. For this reason, all different corticosteroid doses were converted to the equivalent dose for the present study. Methylprednisolone was administered to all patients, with a cumulative dose of 1000 mg (IQR: 485–1340). The median dose of methylprednisolone in patients with ON was 1064 mg (IQR: 1040–1625) and 810 mg (IQR: 460–1260) in patients without ON. Corticosteroids were administered for 47 days (IQR: 40–48) in patients with ON and 19 days (IQR: 12–40) without ON.

All enrolled patients were treated in the ICU and received invasive mechanical ventilation: 18 of 27 patients (67%) by orotracheal intubation and 9 of 27 (33%) by tracheostomy. In addition, all patients received non-invasive oxygen support (Venturi mask and/or nasal cannula) during the hospital stay and/or before dismission from ICU; 22 of 27 patients (81%) were also treated with continuous positive airway pressure (CPAP) devices. During hospitalization, 39% of patients reported thromboembolic complications, and 11% developed sepsis associated with shock.

At follow-up, the median Oxford Hip Score was 38 (IQR: 17–41) in ONFH patients and 48 (46–48) in non-ONFH patients. Among the population study, 86% of patients were able to walk without restriction, whereas 7% reported difficulty walking even one kilometer.

On the EQ-5D-5L questionnaire, 54% of patients reported having no difficulty walking, and 75% reported no problems with self-care. However, 75% of patients did not report musculoskeletal pain, while 36% reported mild discomfort. The health status assessment showed a median score of 75 (IQR: 60–80), indicating a good perception of general health among post-COVID-19 patients.

**Figure 3:**
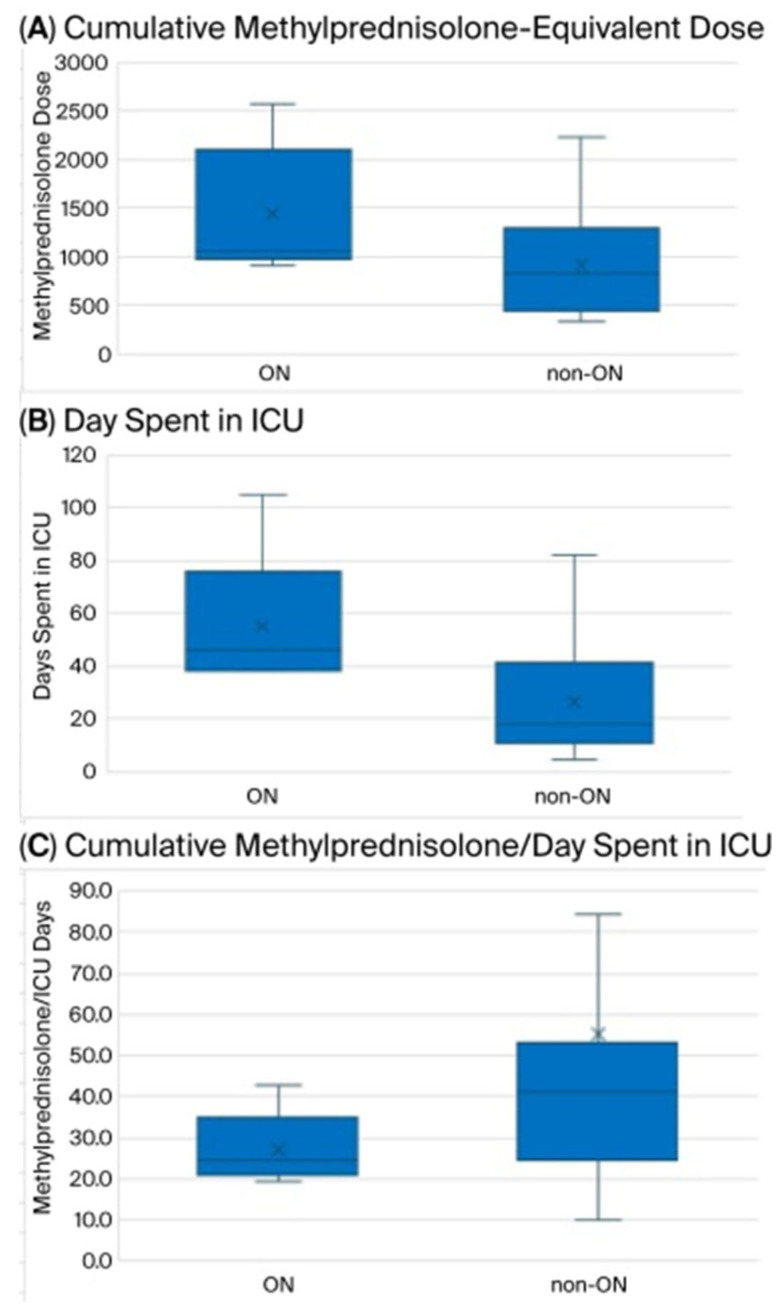
Box plot of the distribution of the main variables. (**A**) Cumulative methylprednisolone-equivalent dose; (**B**) days spent in ICU; (**C**) cumulative methylprednisolone/days spent in ICU. Legend—Lower whisker: minimum, middle whisker: median, upper whisker: maximum; first quartile and third quartile in the blue boxes; the mean value is marked with an “X”.

**Figure 4:**
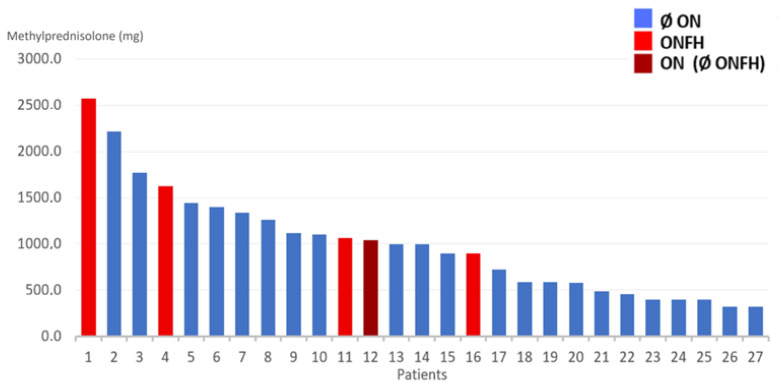
Total dose of methyprednisolone, for every patient. Legend: Ø ON = patient(s) without osteonecrosis; ONFH = patient(s) with osteonecrosis of the femoral head; ON (Ø ONFH) = patients with osteonecrosis but not in the femoral head. In the present study, only one patient showed ON of the talus without ONFH.

**Figure 5:**
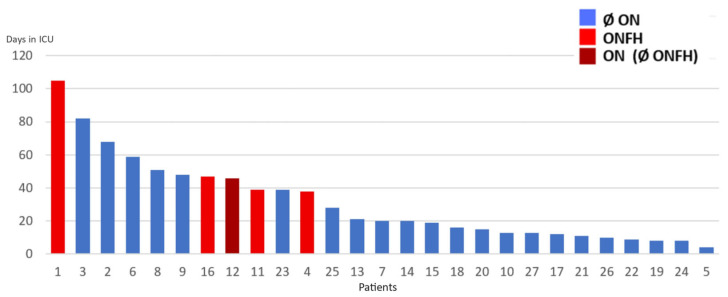
Total days spent in ICU, for every patient. Legend: Ø ON = patient(s) without osteonecrosis; ONFH = patient(s) with osteonecrosis of the femoral head; ON (Ø ONFH) = patients with osteonecrosis but not in the femoral head. In the present study, only one patient showed ON of the talus without ONFH.

**Figure 6:**
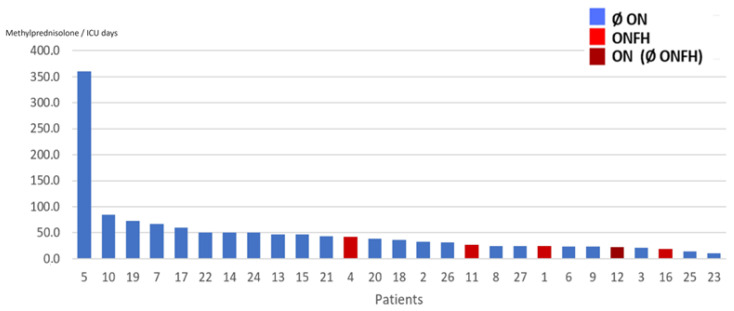
Ratio total methylprednisolone dose/days in ICU, for every patient. Legend: Ø ON = patient(s) without osteonecrosis; ONFH = patient(s) with osteonecrosis of the femoral head; ON (Ø ONFH) = patients with osteonecrosis but not in the femoral head. In the present study, only one patient showed ON of the talus without ONFH.

No missing data was reported in this study. The present study was not designed to compare variables between ONFH and no-ONFH; therefore, formal statistical inference was not performed. Nevertheless, a trend toward more severe disease, higher corticosteroid doses, and lower outcome scores in ONFH patients was observed, as expected. Results are graphically summarized in Figure 3. The visual representation of the raw data concerning each single patient is displayed in Figure 4, Figure 5 and Figure 6. Specifically, results are shown in relation to the total dose of methylprednisolone (Figure 3), days spent in the ICU (Figure 4) and the ratio methylprednisolone to ICU days, as indirect indicators of the peak doses (Figure 5).

After ICU treatment, 19 of 27 patients (70%) were transferred to a rehabilitation clinic, where the mean admission time was 69 days (IQR: 13–109). The remaining 8 of 27 cases (30%) were discharged at home. At the time of writing this paper (2024), none of the enrolled patients had reported or informed the research team about any further symptoms.

## 4. Discussion

In the present study, the prevalence of ONFH during the first pandemic peak was 15% in a high-risk cohort of male patients treated with high doses of corticosteroids for severe COVID-19. Moreover, aside from the hip, MRI of other joints were performed only when symptomatic and resulted in an overall 19% incidence of ON (the sum of ONFH and non-hip ON). These results stress the importance of focused follow-up in patients treated with high-dose corticosteroids and severe COVID-19 to mitigate long-term skeletal complications. More than 2 years after the event, 50% of patients with ONFH were asymptomatic; therefore, clinical assessment alone cannot be considered a reliable tool for detecting ONFH. As early diagnosis is crucial for the outcome of ONFH [12,18,32,33,34,35,36], both high clinical suspicion and MRI are necessary tools in the follow-up of severe COVID-19 treated with high doses of corticosteroids.

The sample size and the inclusion criteria, aimed at selecting a cohort at high risk for ONFH, are limitations of the study, making it difficult to generalize the results and draw robust conclusions about the prevalence of ONFH; the influence of comorbidities and therapies cannot be examined in this study. The absence of ONFH before COVID-19 in this cohort cannot be excluded, as before the admission in the ICU no MRI had been performed with this purpose. Patients with co-pathologies involving cortisone intake or at risk for ONFH were excluded from the study, double-checking both the anamnesis and previous medical charts; however, the authors cannot exclude omitted or reticent anamnesis concerning behaviors at risk like alcoholism and/or drug abuse. The prevalence of ONFH might prove to be overestimated in this sample in comparison to the general COVID-19 population, which also includes mild COVID-19 cases. For these reasons, the reported prevalence cannot be generalized to the overall COVID-19 population. On the other hand, this study selected a homogeneous group of patients during the same pandemic wave and screened for ONFH in both asymptomatic and symptomatic cases.

From early 2020, more researchers paid attention to the association between COVID-19, corticosteroids and ON [1,2,3,4]. This is not surprising, as the association between corticosteroids and ON is well established [8,15,35,36,37,38,39,40,41]. Quantification of the problem drew the attention of the orthopedic community [2,3] to allocate resources for a possible new burden [20,21,22,25]. At the beginning, the most suitable data for a comparison was that derived from the SARS-1 epidemic. As mentioned above, the present study may overestimate the prevalence of ONFH in the COVID-19 population, but even with 15% ONFH occurrence the prevalence in COVID-19 seems to be far below the frequency measured in SARS-1. This pertains particularly to the current work, since the inclusion criteria were based on the data of the 2002–2004 outbreak of SARS-CoV-1 [17,21,28,32,33]. In SARS-1, Guo et al. [19] found ONFH in 40% of a group of young Chinese men treated with a mean cumulative dose equivalent to 4,6 g of methylprednisolone (a very high dose), whereas in the present study the mean dose was 1 g of methylprednisolone. Similarly, Zhao et al. [11] in a meta-analysis of 10 trials with 1137 patients reported a risk ratio of ON 1.57 (95% CI 1.30–1.89, *p* < 0.001) per 5.0 g increase in the cumulative dose of steroids and 1.29 (95% CI 1.09–1.53, *p* = 0.003) for each 10-day increment in treatment duration. The relationship was non-linear (p_non-linear_ < 0.001 and p_non-linear_ = 0.022) and there were no significant differences in the risk of developing ON between male and female patients. Cortisone doses are a crucial variable in the pathogenesis of ON, affecting both the cumulative and peak doses. The incidence of ON in patients treated with high doses of cortisone doses is orders of magnitude greater than in the general population. A British study reported 1.4–3.0/105 inhabitants treated [38]; similarly, a Danish study found 4.2/105 treated individuals [39]. In contrast, patients exposed to high doses of cortisone showed an ON rate of up to 22 to 40% in SARS-1, 16% in systemic lupus erythematosus, 15% in renal transplantation and 7% in bone marrow transplantation, with an overall incidence of 7% (range, 0.3% to 52%) for cumulative doses of prednisone above 10 g [40]. For COVID-19, Takashima et al. screened 26 patients with COVID-19 and reported one case of ONFH (3,8% prevalence) [37]. However, those results are not comparable to the present study since more inclusive criteria were adopted, also including patients with non-severe COVID-19 and without steroid therapy. In a more recent meta-analysis on COVID-19, Muthu et al. [41] found that a cumulative dosage of 4 g and a duration of 15 days were established as the critical threshold for the non-linear dose–response relationship of ON. Moreover, they observed that the risk of ON increased with a pooled OR of 1.16 (95% CI 1.09–1.23, *p* < 0.001) per 2.0 g increase in cumulative dose of cortisone use and with a pooled OR of 1.02 (95% CI 1.01–1.03, *p* < 0.001) per 5 days of increase in the cumulative duration of cortisone [41]. In the present study, there was a trend toward ON with higher cortisone doses (Figure 1), although a formal statistical analysis was not performed due to the study design; moreover, the minimum cortisone dose was lower than in other studies [37,41]. Notably, 2 of 4 (50%) ONFH were ARCO 1 and asymptomatic: those would not have been detected relying on clinical symptoms.

Even if the association between glucocorticoids and ONFH is sound and well established for pathologies related to hematology and rheumatology as well [40,42,43,44], the definition of glucocorticoid associated ONFH is not obvious, as this implies not only the association but also a cause–effect relationship, whereas the exact causative mechanism remains unclear [5,45,46,47,48,49]. Consequently, the expert panel of the Association Research Circulation Osseous working group (ARCO) suggested to change the term “glucocorticoid-induced” to “glucocorticoid associated” ONFH (GA-ONFH) [24]. According to the “multiple hit” theory, the synergy causes include altered bone homeostasis, injured bone cells, impaired blood flow, and suppressed bone cell precursors in susceptible patients [50,51]. Also, the cortisone-induced osteopenia could sustain a vicious circle with the collapse of cancellous bone and consequent vessel occlusion [15,47,51,52]. These ARCO criteria for GA-ONFH [15] were also considered to select the patients for our study. According to the ARCO task force, a dose of 2 g or more administered in <3 months may be considered a threshold for ONFH risk, even if the underlying condition(s) are independently associated with ON [15,19,53,54,55,56,57]. Concerning the interaction between COVID-19, cortison therapy and ONFH, Koutalos et al. [58] found that 3/40 patients on steroids with COVID-19 developed ONFH while 0/40 COVID-19 patients not treated with steroids did not develop ONFH, supporting the independent role of corticosteroids in the development of ONFH after COVID-19. However, Koutalos et al. did not only enroll patients with high dose of steroids; in any case, an independent role of SARS-CoV-2 in ONFH cannot be excluded.

Considering the interaction between SARS-CoV-2 and ONFH, only limited evidence is available. The pathogenesis of corticosteroid-induced ON relies upon reduced blood flow, bone marrow adipocytic hypertrophy leading to sinusoidal compression, venous stasis, obstruction of the arteries and eventually arterial occlusion by fat emboli and lipid-loaded fibrin–platelet thrombi. Other causes include endotoxin-induced disseminated intravascular coagulation, immune reactions and immune complex deposition, immoderately low or high temperatures, and high-impact-related injuries [47,52,59,60]. To date, little data is available concerning the histopathology of ONFH in COVID-19. The histological specimens derive mainly from the femoral heads removed for the implantation of a hip prosthesis because of ONFH. However, the time elapsed between exposure to noxious agents and hip arthroplasty might attenuate some clinical features, such as immune-complex depositions [61]. In 2025, Kuliyeva et al. [62] studied 41 femoral heads after hip arthroplasty in patients with post-COVID-19 ONFH. These authors hypothesized that mast cells might be the main cellular agent in the development of post-COVID-19 ONFH and can be considered a diagnostic sign of the disease. Other studies conducted on soft tissues and organs seem to support the hypothesis of endothelial damage and a pro-thrombotic effect of SARS-CoV-2 [35,61,63,64]. Imagama et al. [65] recently analyzed 5371 registered ONFH patients from a large multicenter Japanese database and compared 20 patients (32 hips) who developed ONFH after COVID-19 with a non-COVID-19 ONFH group. The authors concluded that patients in the COVID-19 group had lower corticosteroid use, higher prevalence in men, and higher rate of early flattening of the weight-bearing surface, underscoring the potential impact of COVID-19-related thrombotic pathways. Therefore, clinicians should perform appropriate investigations as early as possible in COVID-19 patients presenting with musculoskeletal symptoms, even when corticosteroid use is limited. Similar results have also been found in SARS, but there are many points which make SARS-1 and COVID-19 not comparable. Concerning the therapy, in SARS-1 the doses of corticosteroids used were higher than COVID-19. Other variables such as the exposure to oxygen fluxes, auxiliary medical therapy and the severity of the disease itself might influence the results [31,66], but details are scarcely reported or not available. Concerning the RNA-viruses and the immune response of the host (humans), both are continuously evolving [35,61,62,67] through the interaction of different variants of SARS-CoV-2 and vaccinations, and this might also play a role in the pathogenesis of ONFH [21,68,69,70,71]. In this regard, this study presents a unique data-set relative to the first pandemic wave and without vaccination (at that time, no vaccine was available for clinical practice) [21,68,69,70,71].

The underlying co-pathologies and their medical treatment could have influenced the outcomes. The individual pathologies encountered in this study are listed in the section “Comorbidities and risk factors” in Table 1. Although the ON group tended to have a higher percentage of pathologies represented in the ON group, in the present study it is not possible to draw conclusions about comorbidities and risk factors as it was not designed for this purpose, and the inclusion criteria may have selected more severely affected patients.

## 5. Conclusions

A high-risk cohort of patients with severe COVID-19 treated with high doses of corticosteroids had a 15% prevalence of ONFH following selective MRI screening, and two years after the event, 50% of ONFH cases were asymptomatic. Relying solely on clinical evaluation in these patients might risk underestimating ONFH, thus potentially influencing treatment and outcomes. Moreover, other joints might develop ON. The association between severe COVID-19, high doses of corticosteroids and ONFH suggests the need for focused clinical evaluation, including magnetic resonance imaging.

## Figures and Tables

**Table 1:** Demographic and clinical characteristics of patients with and without osteonecrosis. In brackets are the percentage and range, according to data.

		Osteonecrosis	
	Total	No	Yes
	*N* = 27	*N* = 22	*N* = 5
ANAMNESIS			
Age at COVID-19 disease	54 (50–57)	53.5 (51–57)	54 (48–55)
Diabetes	7 (26)	5 (23)	2 (40)
Hypertension	13 (48)	8 (36)	5 (100)
Dyslipidemia	4 (15)	2 (9)	2 (40)
Obesity	6 (22)	4 (18)	2 (40)
Smoker	2 (7)	2 (9)	0 (0)
Others	0 (0)	0 (0)	0 (0)
Days in ICU	21.0 (13.0–48.0)	18.5 (12.0–40.0)	47.0 (40.0–48.0)
Days of hospitalization	67 (34–92)	62.5 (28–91)	67 (64–128)
Methylprednisolone [1]	27 (100)	22 (100)	5 (100)
Days of methylprednisolone	21 (13–48)	18.5 (12–40)	47 (40–48)
Cumulative dose of methylprednisolone	1000 (485–1340)	810 (460–1260)	1064 (1040–1625)
FUNCTIONAL DATA			
Walking distance			
Only at home	2 (7)	1 (5)	1 (20)
1 km	1 (4)	0 (0)	1 (20)
Unlimited	24 (89)	21 (95)	3 (60)
Presence of musculoskeletal pain			
Absent	21 (78)	20 (91)	1 (20)
Present at rest	2 (7)	1 (5)	1 (20)

**Table 2:** MRI findings of the patients with ON, with the ARCO classification for those with ONFH, the cumulative dose of corticosteroids equivalent, time from diagnosis to first hospitalization, and length of hospitalization.

Patient Reference Number	ONFH Laterality	ARCO Classification	Additional ON Sites	Cumulative Dose of Methylprednisolone (mg)	Time of Diagnosis from First Hospitalization (Months)	Length of Hospitalization (Days)
1	R+L	R: 4 L: 4	Proximal Humerus (R+L), Distal Femur (R+L) and Talus (R+L)	2569.8	4.2	128
4	R+L	R: 1A L: 1B	-	1625.0	28.2	58
11	R	2C	-	1064.0	27.7	65
12	-	-	Talus (R)	1040.0	28.8	67
16	L	2B	-	900	31.7	148

R = right side, L = Left side.

## Data Availability

The data that supports the findings of this study are available from the corresponding author, upon reasonable request.

## Abbreviations

The following abbreviations are used in this manuscript:

ARCO	Association Research Circulation Osseous
COVID-19	Coronavirus Disease of 2019
ICU	Intensive care unit
MRI	Magnetic resonance imaging
ON	Osteonecrosis
ONFH	Osteonecrosis of femoral head
SARS-1	Severe acute respiratory syndrome 1 (due to SARS-CoV-1)
SARS-CoV-2	Severe acute respiratory syndrome coronavirus 2
